# Use of molecular typing to investigate bacterial translocation from the intestinal tract of chlorpyrifos-exposed rats

**DOI:** 10.1186/s13099-016-0129-x

**Published:** 2016-11-05

**Authors:** Claire Joly Condette, Bertin Elion Dzon, Farida Hamdad, Maurice Biendo, Véronique Bach, Hafida Khorsi-Cauet

**Affiliations:** 1 Laboratoire PeriTox UMR I 01, Faculty of Medicine, Centre Universitaire de Recherche Scientifique, Université de Picardie Jules Verne, Avenue René Laёnnec, 80054 Amiens cedex 1, France; 2 Laboratoire LNPC EA4666, Faculty of Medicine, Centre Universitaire de Recherche Scientifique, Université de Picardie Jules Verne, Avenue René Laёnnec, 80054 Amiens cedex 1, France; 3 Laboratoire de Bactériologie, Centre Hospitalier Universitaire Amiens Picardie, Avenue René Laёnnec, Salouёl, 80054 Amiens cedex 1, France

**Keywords:** Rat, Bacterial translocation, Chlorpyrifos, In utero, Lactational period, Intestinal permeability, Molecular typing

## Abstract

**Background:**

Human are confronted on a daily basis with contaminant pesticide residues in food, water and other components of the environment. Although the digestive system is the first organ to come into contact with food contaminants, very few data are available on the impact of low-dose pesticide exposure during the in utero and postnatal periods on intestinal bacterial translocation (BT). Previous studies have revealed that chlorpyrifos (CPF) exposure is associated with intestinal dysbiosis and the contamination of sterile organs. Here, molecular typing was used to investigate intestinal bacterial translocation in rats exposed to chlorpyrifos in utero and during lactation. The translocated bacteria were profiled, and CPF tolerance and antibiotic resistance traits were determined.

**Methods:**

A total of 72 intestinal segments and extra-intestinal organs were obtained from 14 CPF-exposed rats. The samples were cultured to isolate bacterial strains that had tolerated treatment with 1 or 5 mg CPF/kg bodyweight/day in vivo. Strains were identified using matrix-assisted laser desorption/ionization (MALDI) Biotyper. The disk diffusion method was used to determine the antibiotic susceptibility. The isolates were genotyped with PCR assays for the enterobacterial repetitive intergenic consensus sequence and random amplification polymorphic DNA.

**Results:**

Bacterial translocation was confirmed for 7 of the 31 strains (22.6 %) isolated from extra-intestinal sites. Overall, the most prevalent bacteria were *Staphylococcus aureus (*55.5 % of the 72 intestinal and extra-intestinal isolates), *Enterococcus faecalis* (27.7 %) and *Bacillus cereus* (9.8 %). 5 % of the *S. aureus* isolates displayed methicillin resistance. Seventy two strains were identified phenotypically, and seven translocated strains (mainly *S. aureus*) were identified by genotyping. Genotypically confirmed translocation was mainly observed found in pesticide-exposed groups (6 out of 7).

**Conclusion:**

BT from the intestinal tract colonized normally sterile extra-intestinal organs in CPF-exposed rats. Our findings validate the use of molecular typing for the assessment of intestinal BT in CPF-exposed rats during critical periods of development.

## Background

Highly toxic organophosphorus compounds are the main constituents of many of the agricultural, industrial, and residential insecticides used worldwide. The acute toxicity associated with high doses of organophosphorus pesticides is caused by inhibition of acetylcholinesterase and the resulting increase in synaptic acetylcholine levels [1]. However, there is substantial evidence to show that this mechanism alone cannot account for the wide range of harmful effects associated with organophosphorus pesticides—especially when the level of exposure is below the threshold for acute toxicity [2].

Chlorpyrifos (CPF) is a major organophosphorus insecticide. It is used to treat fruit and vegetable crops and exists as chlorpyrifos ethyl [the most toxic form: *O*,*O*-diethyl-*O*-(3,5,6-trichloro-2-pyridyl)-phosphorothionate; chemical formula: C_9_H_11_Cl_3_NO_3_PS] and chlorpyrifos methyl.

Although CPF residues can be found in cereals, fruit, vegetables and (potentially) meat and drinking water, few studies have focused on the compound’s putative impact on the digestive tract. Tirelli et al. [3] have shown that CPF increases membrane permeability in an enterocyte cell culture model. Furthermore, Joly Condette et al. [4] showed for the first time that low-dose chlorpyrifos exposure in vivo causes morphological changes in the intestinal epithelium, alters intestinal permeability and increases bacterial translocation (BT, as detected by culture-based methods). In rats, this phenomenon is associated with (and probably caused by) failure of the intestine’s barrier function and an imbalance of in the intestinal microbiota [5].

The intestinal microbial community is a complex ecosystem that influences the host’s physiology in many ways. The bacterial count in the caecum and colon reaches values of 10^12^/g in the feces, whereas nearby portal blood, mesenteric lymph nodes (MLNs) and organs (like kidney, liver, spleen) are usually sterile. This illustrates the efficacy of this intestinal barrier.

Berg has defined BT as the migration of microorganisms and their toxins from the intestinal lumen to sterile organs such as the MLNs, blood, and abdominal organs [6]. BT can lead to a local inflammatory response and a potential increase in intestinal permeability, which in turn can lead to systemic infection and multiple organ failure [7]. Arnold and Brody were the first to confirm the harmfulness of BT in animals [6, 8]. In humans, some degree of BT is observed in Crohn’s disease, neutropenia, hemorrhagic shock, necrotizing enterocolitis, delayed sepsis, systemic inflammatory response syndrome, and multi-organ failure [9, 10]. Reduced blood flow in the gut, trauma, chronic inflammation, and immunosuppression are all factors that enhance BT [11].

BT from the intestine is most commonly detected by measuring the presence of viable bacteria in extra-intestinal target tissues. This reflects not only the integrity of the mucosal barrier function but also the numbers and types of microbes in the lumen. Translocation may occur by three mechanisms: (i) through microfold cells [12] (a normal processing pathway that is especially active during specific life periods, including the neonatal period) [13–15]; (ii) through intestinal epithelial cells (a major pathway after cellular injury such as that caused by cytotoxic drugs) [16], and (iii) after bacterial overgrowth and/or impairment of host defenses [7].

Since the digestive system appears to be a target for CPF and the only study on BT in this context was based on conventional microbiological tests, we repeated our original study by using molecular techniques to investigate intestinal BT (to the liver, spleen, MLNs, Peyer’s patches, and kidney) in CPF-exposed rats, identify specific bacterial strains, and confirm the bacteria’s intestinal origin.

Lastly, we sought to determine whether prolonged, low-dose exposure to CPF for different periods of time promotes the emergence of antibiotic-resistant bacterial strains in rats.

## Methods

### Experimental animals and housing

All animal experiments were approved by the Animal Care and Use Committee at Jules Verne University of Picardy (Amiens, France: reference #2011/A/1).

The laboratory animals used in this study (15 female and 5 male Wistar Hannover rats; age on delivery: 8 weeks; body weight range: 215–300 g) were obtained from Janvier Labs (Le Genest-Saint-Isle, France). The rats were allowed to acclimatize to the laboratory for at least one week period prior to the experiment. The animals were housed in plastic cages and fed a diet of standard rat pellets. Water was provided ad libitum. The protocol has been described previously [4].

### Schedule for CPF treatment

After the acclimation period, female rats were mated with males (two females per male). Once a positive smear was observed, dams received daily doses of the respective treatment from days 0 to 21 (D21, the day of weaning) by oral gavage. Three exposure groups were studied: the rats in the CPF0 (control) group received 1 mL/kg bodyweight (BW) of rapeseed oil; the rats in the CPF1 group received 1 mg/kg BW/day of CPF in rapeseed oil, and the rats in the CPF5 group received 5 mg/kg BW/day of CPF in rapeseed oil. The rats in the litters were studied at two time points: firstly at weaning (after having been exposed to CPF in the dam’s milk (D21: CPF0, n = 7; CPF1, n = 8, and CPF5, n = 6) and secondly in young adulthood (after the young rats had been gavaged with CPF individually from weaning to the age of 60 days (D60: CPF0, n = 5; CPF1, n = 7, and CPF5, n = 8).

### Definition of BT

In an individual rat, BT was defined as the presence of the same bacterial species [with identical or closely related enterobacterial repetitive intergenic consensus (ERIC2) sequence and random amplification polymorphic DNA (RAPD) patterns in PCR assays] in both the intestinal segments and the normally sterile extra-intestinal organs.

### Tissue cultures

The tissue specimens obtained from each rat were separately mashed, dilacerated, and homogenized in 9 mL of Ringer’s solution in a sterile lab blender bag (Stomacher^®^, Seward Medical Ltd, Worthing, UK). Next, 1 mL of the homogenate was diluted to one tenth of its original concentration (1:10). 100 μL volume of each diluted homogenate was inoculated onto aerobic and anaerobic Columbia blood agar, chocolate agar, and Chapman agar plates (bioMérieux, Marcy l’Etoile, France), which were incubated at 35 ± 2 °C in 5 % CO_2_ for 24–72 h. Isolates grown from samples (i.e. each colony) were numbered and identified on the basis of their characteristic colonial and microscopic appearances and matrix-assisted laser desorption/ionization time-of-flight mass spectrometry (MALDI–TOF-MS) pattern.

### Definition of duplicates

Rats that produced more than one positive sample for the same bacterium with the same phenotypic expression of antibiotic susceptibility were assessed only once. Hence, duplicates were excluded from the study.

### Bacterial identification by MALDI Biotyper

A MALDI–TOF-MS system (Autoflex III, Bruker Daltonics, Billerica, MA, USA) was used to examine each unique colony of microorganisms isolated by culture, according to a previously described procedure [17–19]. In brief, samples were prepared with absolute ethanol, and 1 μL of matrix solution (2,5-dihydroxybenzoic acid 50 mg/mL, 30 % acetonitrile, 0.1 % trifluoroacetic acid) was added. The analytical data were processed with Bruker Biotyper software (version 2.0, Bruker Daltonics), as described previously [20]. Identifications were performed in duplicate, according to the manufacturer’s instructions.

### Antibiotic susceptibility testing

As recommended by the *Comité de l’Antibiogramme de la Société Française de Microbiologie* [21], the disk diffusion method (with Mueller–Hinton agar at 35 ± 2 °C for 24 h) was used to test the bacterial isolates’ antibiotic susceptibility. *Staphylococcus aureus* and *Staphylococcus warneri* were tested for susceptibility to the following 20 antibiotics: streptomycin (10 µg), kanamycin (30 μg), gentamicin (30 μg), tobramycin (30 μg), fosfomycin (50 μg), doxycycline (30 μg), trimethoprim–sulfamethoxazole (1.25 + 23.75 μg), erythromycin (Er: 15 μg), lincomycin (L: 15 μg), pristinamycin (PT: 15 μg), rifampin (30 μg), ofloxacin (OFX: 5 μg), vancomycin (30 μg), teicoplanin (30 μg), linezolid (10 μg), fusidic acid (10 μg), benzylpenicillin (PEN: 6 μg), oxacillin (OXA: 5 μg), cefoxitin (FOX: 30 μg), and moxalactam (30 μg). *Enterococcus f*ae*calis* was tested for susceptibility to ampicillin (10 μg), kanamycin (1000 μg), gentamicin (500 μg), streptomycin (500 μg), Er (15 μg), L (15 μg), PT (15 μg), teicoplanin (30 μg), vancomycin (30 μg), trimethoprim–sulfamethoxazole (1.25 + 23.75 μg), and linezolid (10 μg). The isolates were classified as susceptible (S), intermediate (I) and resistant (R) on the basis of established breakpoint values [21].

### Determination of the bacterial strains’ clonality

Total nucleic acids were extracted from bacteria grown on Columbia agar supplemented with 5 % sheep blood incubated at 35 ± 2 °C for 18–24 h. Each a bacterial colony was suspended in 300 μL of distilled water, transferred to 1.5 μL microfuge tubes, and incubated at 95 °C for 10 min. The cell suspension was incubated in a bath-type ultrasonic sonicator (Gen-Probe; bioMérieux, Craponne, France) for 15 min and then centrifuged at 13,500 rpm for 10 min. A 400 μL aliquot of eluate was carefully transferred to a new sterile Eppendorf tube and stored at −20 °C until use in the polymerase chain reaction (PCR) assays. PCR was performed in a final volume of 50 μL of the corresponding ready-to-use Master Mix buffer from the TopTaq Master Mix Kit (Qiagen, Venlo, Netherlands). Each reaction mixture contained 25 μL of Master Mix buffer, 20 μM of ERIC2 primer (5′-AAG-TAA-GTG-ACT-GGG-GTG-AGC-G-3′) [22] or 20 μM of RAPD1 PRIMER (5′-GCT-TGG-GTG-AGA-ATT-GCA-GG-3′) [23], with 1.5 μL of DNA for ERIC2 and 2 μL of DNA for RAPD) used as a template, and 5 μL of colored loading buffer for each PCR.

The amplification conditions for ERIC2-PCR were as follows: 7 min at 94 °C, followed by 45 cycles of 1 min at 94 °C, 1 min at 45 °C, and 2 min at 72 °C, with a final extension of 7 min at 72 °C. The conditions for RAPD-PCR were as follows: 3 min at 94 °C, followed by 45 cycles of 1 min at 94 °C, 1 min at 36 °C, and 5 min at 72 °C, with a final extension of 7 min at 72 °C. All PCRs were carried out in a GeneAmp 2400 thermal cycler (Perkin Elmer Inc., Waltham, MA, USA).

PCR products were resolved by electrophoresis on a 1 % agarose gel in Tris–acetate-EDTA (Sigma-Aldrich, USA) containing 0.5 μg/mL of ethidium bromide. SmartLadders^®^ (200–10 kb; Eurogentec, Seraing, Belgium) were used as molecular weight markers. The gel profiles were photographed with an ultraviolet light transilluminator (ChemiDoc Touch Imaging System, Bio-Rad Laboratories, Marnes-la-Coquette, France) and processed with Image Lab software (version 5.2.1, Bio-Rad).

### Statistical analysis

All statistical analyses were performed with R software (version 3.1.0; Lucent Technologies, Murray Hill, NJ, USA).

The frequency of BT was quoted with its 95 % confidence interval (CI, calculated using the “minlike” method) [17]. A mixed logistic regression model was used to determine the relationships between the likelihood of BT on one hand and exposure and age on the other. The odds ratio (OR) [95 % CI] for BT was also calculated. The threshold for statistical significance was set to p < 0.05.

## Results

### Microbiological tests

Out of a total of 126 cultured tissue samples, 46 were negative and 80 were positive. After eliminating duplicates (see below), 72 positive samples (40 intestinal fragments and 32 samples from extra-intestinal organs) were studied.

When considering the 72 samples from various sites (Table 1), *S. aureus* was the most commonly identified organism (n = 40 positive samples, 55.5 %), followed by *E. faecalis* at (n = 20, 27.7 %), *Bacillus cereus* (n = 7, 9.8 %), *S. warneri* (n = 3, 4.2 %) and *Micrococcus luteus* (n = 2, 2.8 %).

**Table 1:** Distribution of bacterial strains by sample type (n = 72)

Samples					
Strains	Intestinal segments	No	Extra-intestinal organs	No	Total
*Staphylococcus aureus* (n = 40)	Caecum Colon Ileum	13 10 5 – –	Kidney Liver Peyer’s patch Spleen Adipose tissues Mesenteric lymph node	4 3 2 1 1 1	
	Subtotal	28		12	40
*Enterococcus f*ae*calis* (n = 20)	Colon Caecum	7 3 – –	Kidney Peyer’s patch Liver Mesenteric lymph node	4 3 2 1	
	Subtotal	10		10	20
*Bacillus cereus* (n = 7)	Ileum	2	Kidney	5	
	Subtotal	2		5	7
*Staphylococcus warneri* (n = 3)			Liver Kidney	2 1	
	Subtotal	0		3	3
*Micrococcus luteus* (n = 2)			Liver Adipose tissues	1 1	
	Subtotal	0		2	2
Total		40		32	72

### Antibiotic susceptibility

The susceptibility results showed that 38 of the 40 *S. aureus* isolates (95 %) were methicillin-susceptible, and 2 (5 %) were resistance to methicillin and OFX (i.e. methicillin-resistant *S. aureus*, MRSA).

All *S. warneri*, *B. cereus* and *M. luteus* isolates were susceptible to the antibiotics tested with the *Staphylococcus* genus, except for two strains of *S. warneri* that were resistant to fusidic acid and Er.

For *E. faecalis*, all isolates were susceptible to ampicillin, vancomycin, teicoplanin and trimethoprim–sulfamethoxazole, and all were resistant to L. The *E. faecalis* isolates showed a low-to-moderate level of resistance to Er [11 out of 20 were susceptible (55 %) and 9 (45 %) were resistant] and PT [14 were susceptible (70 %) and 6 were resistant (30 %)].

### Genotyping

We isolated 72 strains of *S. aureus*, *E. faecalis* and *B. cereus* from samples collected from 14 rats. The strains were compared with each other by using ERIC2-PCR and RAPD-PCR assays. The other species (*S. warneri* and *M. luteus*) found in samples from three rats (animal ID numbers: 19.9, 15.8 and 3.8) were not typed because of their lower prevalence.

The *S. aureus* isolates were compared in an ERIC2-PCR analysis (n = 40). A total of 28 distinct ERIC2-PCR types were detected (referred to as E1 through E28). ERIC2-PCR types E21, E22, and E25 included four isolates each (isolates 24, 25, 38 and 40 for E21; isolates 26, 27, 34, and 35 for E22, and isolates 30–33 for E25). Within each profile, the isolate were genetically related. Two isolates each were found for E9 (isolates 9 and 10), E15 (isolates 16 and 17), and E16 (isolates 18 and 20).

**Fig. 1:**
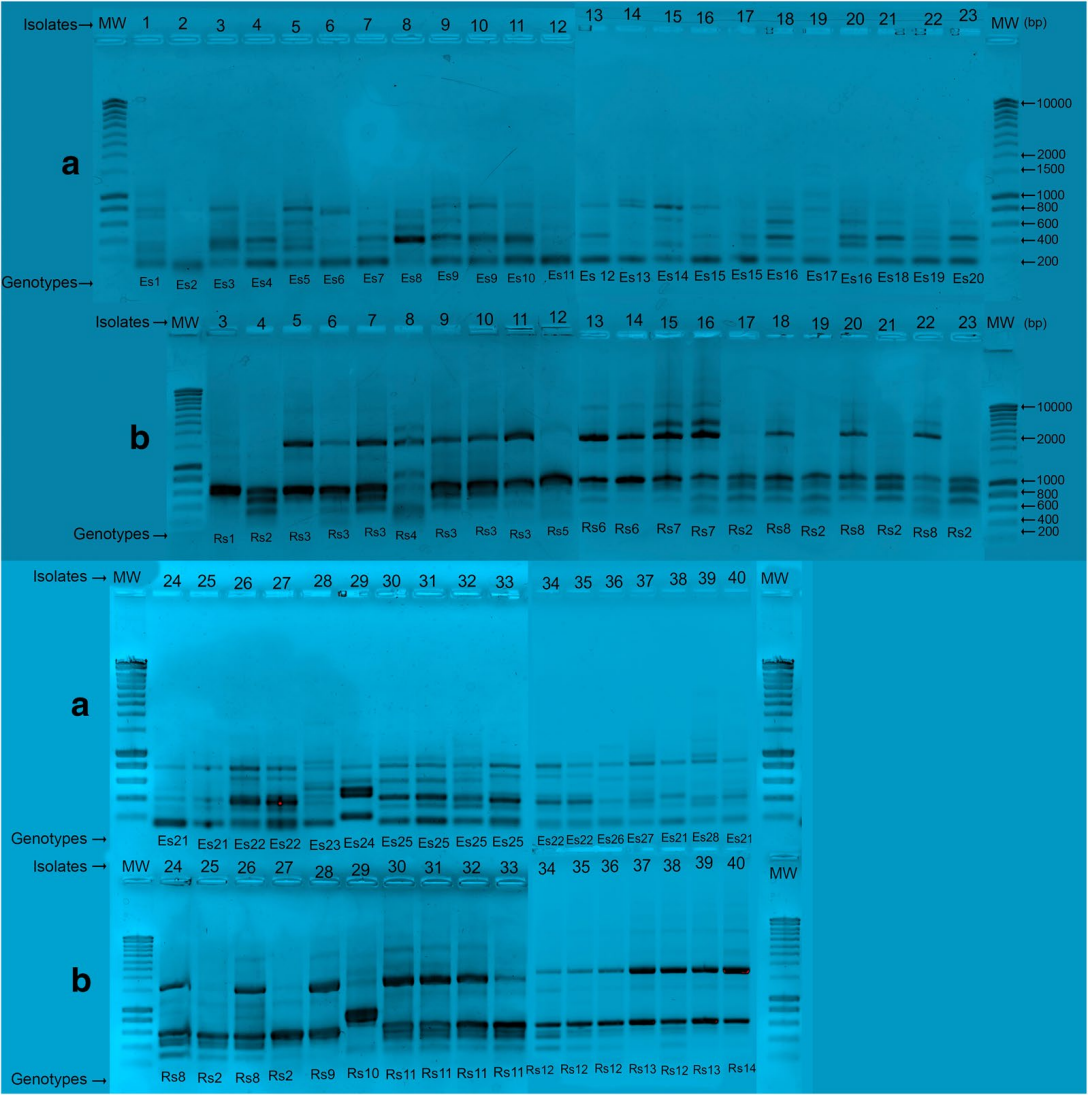
**a** ERIC-PCR profiles for *S. aureus*: *Lines* 1–40 isolate numbers (*above* gels) and pattern types (*below* gels). Molecular weights (MW) are expressed in SmartLadder base pairs (bp). The 40 isolates were differentiated into 28 distinct ERIC-PCR patterns (E1–E28). **b** RAPD-PCR profiles for *S. aureus*: *Lines* 3–40 isolate numbers (*above* gels), and pattern types (*below* gels). Molecular weights (MW) are expressed in SmartLadder base pairs (bp). The 38 isolates were differentiated into 12 distinct types (R1–R12)

The remaining profiles included one isolate each. In an RAPD-PCR analysis, the 40 *S. aureus* isolates were differentiated into 12 distinct types (referred to as R1 through R12). The nucleic acids of two isolates (1 and 2) could not be studied because the strain was not sufficiently viable. The following RAPD types had genetically identical isolates within each profile: R2 consisted of seven identical isolates (isolates 4, 17, 19, 21, 23, 25 and 27), R3 consisted of six identical isolates (5–7 and 9–11), R8 consisted of five identical isolates (18, 20, 22, 24, and 26), R11 consisted of four identical isolates (30–33), R12 consisted of three identical isolates (34–36), R10 consisted of three identical isolates (37–39), R6 consisted of two identical isolates (13 and 14), and R7 consisted of two identical isolates (15 and 16) (Fig. 1a, b).

**Fig. 2:**
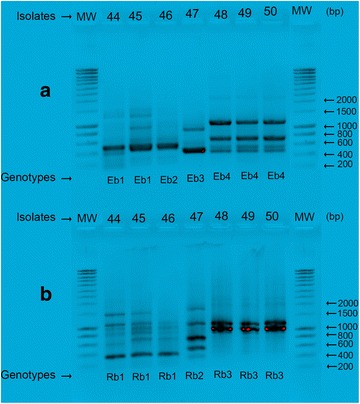
**a** ERIC-PCR profiles for *B. cereus*: *Lines* 44–50 isolate numbers (*above* gels) and pattern types (*below* gels). The seven isolates were differentiated into four distinct types (E1–E7). **b** RAPD-PCR profiles for *B. cereus*: *Lines* 44–50 isolate numbers (*below* gels). The seven isolates were differentiated into three distinct types (R1–R3)

ERIC2- and RAPD-PCRs were then used to evaluate the relatedness of *B. cereus isolates* (n = 7). Four patterns were generated with the ERIC primer (denoted by E1 through E4): E4 included four isolates (48–50), E1 included two isolates (44 and 45), and one isolate each was found for patterns E2 (isolates 46) and E3 (isolates 47). RAPD-PCR revealed three profiles (designated as R1 through R3): four genetically indistinguishable isolates were found for the profiles R1 (44–46) and R3 (48–50). The R2 pattern was considered to be an unrelated type (isolate 47) (Fig. 2a, b).

ERIC2-PCRs of *E. faecalis* isolates (n = 20) revealed ten patterns (denoted by E1 through E10). Pattern E6 comprised five isolates (58–61 and 63), pattern E9 comprised four isolates (65, 66, 71, and 72), pattern E10 comprised three isolates (67–69), and pattern E2 comprised two isolates (64 and 70). Within each profile, all the isolates were genetically identical. The remaining patterns (E1, E5 and E7) consisted of one isolate each.

**Fig. 3:**
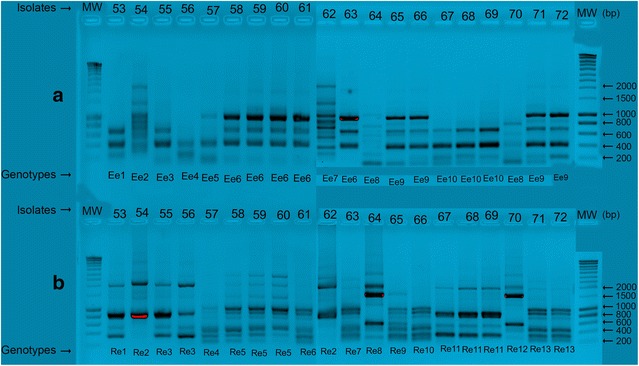
**a** ERIC-PCR profiles for *E. f*ae*calis*: *Lines* 53–72 isolate numbers (*above* gels) and pattern types (*below* gels). Molecular weights (MW) are expressed in SmartLadder base pairs (bp). The 20 isolates were differentiated into ten distinct types (E1–E10). **b** RAPD-PCR profiles for *E. f*ae*calis*: *Lines* 53–72 isolate numbers (*above* gels) and pattern types (*below* gels). Molecular weights (MW) are expressed in SmartLadder base pairs. (bp). The 20 isolates were differentiated into 13 distinct type (R1–R13)

RAPD-PCRs of *E. faecalis* generated 13 distinct profiles (denoted by R1 through R13). Three isolates each were found for the patterns R5 (58, 59 and 60) and R11 (67, 68 and 69). Patterns R2 (isolates 54 and 62), R3 (isolates 55 and 56), and R13 (isolates 71 and 73) comprised two isolates each. Within each profile, all the isolates were genetically identical. The remaining profiles included one genetically unrelated isolate each (Fig. 3a, b).

### Assessment of genetic groups

The combination of *S. aureus* isolates obtained with ERIC2-PCR and RAPD-PCR enabled us to define 34 genetic groups (Ggs, denoted by A through Z and AA through JJ). Gg DD (patterns E25-R11) included four isolates (30–33), whereas two isolates each were found for GgI (9 and 10 for patterns E9-R3), GgQ (18 and 20 for patterns E16-R8), and Gg EE (38 and 40 for patterns E21-R12). Within each Gg, all the isolates were genetically identical. The other Ggs comprised one isolate each and were considered to be unrelated.

The combined typing results for *B. cereus* strains revealed four Ggs (denoted by A through D). GgA included two isolates (44 and 45) and GgD consisted of three isolates (48–50). Within each group, these isolates were genetically similar to each other. One isolate each was determined for GgB (46) and GgC (47); these isolates were considered to be unrelated.

Combining the ERIC2-PCR and RAPD-PCR patterns for *E. faecalis* gave 16 Ggs (referred to as I through XVI). The strains in each group (isolates 58–60, Gg VI), (isolates 67–69, Gg XIII), and (isolates 71 and 72, Gg XVI) were genetically related. The remaining groups included one isolate each and showed polyclonal heterogeneity.

### Bacterial translocation

**Fig. 4:**
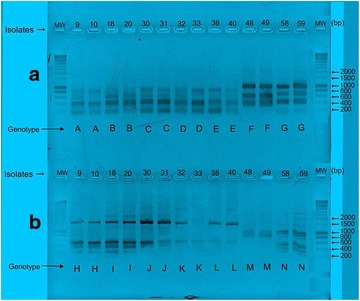
Translocated isolates. **a** ERIC-PCR profiles for *S. aureus* [*lines* (9, 10), (18, 20), (30, 31), (32, 33), (38, 40)]; *B. cereus* (lines 48, 49), and *E. f*ae*calis* (*lines* 58, 59): isolate numbers (*above* gels) and pattern types (*below* gels). Molecular weights (MW) are expressed in base pairs (pb). **b** RAPD-PCR profiles for *S. aureus* [*lines* (9, 10), (18, 20), (30, 31), (32, 33), (38, 40)]; *B. cereus* (48, 49); and *E. f*ae*calis* (58, 59): isolate numbers (*above* gels) and pattern types (*below* gels). Seven isolates (as defined genotypically) were found to have translocated

Translocation was observed in 7 of the 31 CPF-exposed strains (22.6 %, with a 95 % CI of [7–36 %]; Table 2). Five were detected at D21 and 2 were detected at D60; one belonged to the CPF0 group, 3 (42.9 %) belonged to the CPF1 group and the remaining 3 (42.9 %) belonged to the CPF5 group (Fig. 4a, b).

**Table 2:** Isolates for which bacterial translocation was confirmed (7 out of 31 isolates)

Bacterial strains	Rat ID number	Age (days)	CPF group	Isolates No	Intestinal segments	Extra-intestinal organs
*Staphylococcus aureus* (n = 10)	12.6	D21	CPF0	9	Caecum	–
				10	–	Adipose tissues
	18.6	D21	CPF1	18	Colon	–
				20	–	Kidney
	6.14	D60	CPF1	30	Caecum	–
				31	–	Peyer’s patch
	4.4	D21	CPF5		Caecum	–
					–	Kidney
	4.7	D60	CPF5	38	Caecum	–
				40	–	Kidney
*Bacillus cereus* (n = 2)	13.2	D21	CPF5	48	Ileum	–
				49	–	Kidney
*Enterococcus f*ae*calis* (n = 2)	15.8	D21	CPF1	58	Caecum	–
				59	–	Liver

Prevalence: 22 % (7 out of 31)

Translocation of *S. aureus* was evidenced in 5 of the 40 caecum and colon samples (12.5 %); translocation to the kidney: n = 3; translocation to adipose tissues: n = 1; translocation to Peyer’s patches: n = 1

Translocation of *B. cereus* was evidenced in 1 of 7 ileum samples (14.2 %); translocation to adipose tissues

Translocation of *E. f*ae*calis* was evidenced in 1 of 20 caecum samples (5 %); translocation to the liver

Of the 24 BT-free rats, 8 (33.4 %) belonged to the CPF0 group, 10 (41.6 %) belonged to the CPF1 group, and 6 (25 %) belonged to the CPF5 group; 16 (66.6 %) were detected at D21 and 8 (33.4 %) were detected at D60 (Table 3). Translocation increase with CPF exposure (OR [95 % CI] = 1.25 [0.22–7.15]), although this was not statistically significant (*p* = 0.7981). Furthermore, translocation was more likely on D21 than on D60 (OR [95 % CI] = 1.25 [0.22–7.15]); again, the OR was not statistically significant (*p* = 0.7981).

**Table 3:** Isolates for which bacterial translocation was not confirmed (24 out of 31 isolates)

Bacterial strains	Rat ID number	Age (days)	CPF group	Isolates No	Intestinal segments	Extra-intestinal organs
*Staphylococcus aureus* (n = 30)				1	Ileum	–
	14.7	D21	CPF0	2	Caecum	–
				3	–	Liver
	19.9	D21	CPF0	4	Caecum	–
				5	–	Liver
				6	Ileum	–
	12.6	D21	CPF0	7	Ileum	–
				8	Colon	–
	19.4	D60	CPF0	11	Colon	–
				12	Caecum	–
				13	Ileum	–
	19.5	D60	CPF0	14	Colon	–
				15	Caecum	–
				16	–	Peyer’s patch
	18.6	D21	CPF1	17	Ileum	–
				19	–	Spleen
				21	Colon	–
	15.8	D21	CPF1	22	Caecum	–
				23	–	Liver
				24	Caecum	–
	24.7	D21	CPF1	25	–	Kidney
				26	–	Spleen
				27	Colon	–
	8.13	D60	CPF1	28	Caecum	–
				29	–	Kidney
				34	Ileum	–
	13.2	D21	CPF5	35	Colon	–
				36	Caecum	–
	4.7	D60	CPF5	37	Colon	–
				39	–	Mesenteric lymph node
*Enterococcus f*ae*calis* (n = 18)	12.6	D21	CPF0	53	Colon	–
	19.14	D60	CPF0	54	–	Liver
				55	Colon	–
	18.6	D21	CPF1	56	Colon	–
	15.8	D21	CPF1	57	Colon	–
	24.7	D21	CPF1	60	Colon	–
				61	–	Kidney
				62	Caecum	–
				63	Caecum	–
	6.14	D60	CPF1	64	–	Peyer’s patch
				65	–	Peyer’s patch
				66	–	Peyer’s patch
	3.8	D21	CPF5	67	Colon	–
	4.4	D21	CPF5	68	–	Kidney
				69	–	Kidney
	4.7	D60	CPF5	70	Colon	–
				71	–	Mesenteric lymph node
				72	–	Kidney
*Bacillus cereus* (n = 5)	19.9	D21	CPF 0	44	Ileum	–
	15.8	D21	CPF1	45	–	Kidney
				46	–	Kidney
	8.13	D60	CPF1	47	–	Kidney
	13.2	D21	CPF5	50	–	Kidney

The bacteria that had translocated from the intestinal tract and were detected in the extra-intestinal samples were distributed as follows: 5 of the 40 *S. aureus* isolates (12.5 %) from the caecum (n = 4) or the colon (n = 1) had translocated to the kidneys (n = 3), adipose tissue (n = 1) or Peyer’s patches (n = 1). One of the 20 *E. f*ae*calis* isolates (5 %) from the caecum had translocated to the liver, and one of the 7 *B. cereus* isolates (14.2 %) from the ileum had translocated to the kidneys (Table 2). The typing assay results confirmed these data. By combining the ERIC2-PCR and RAPD-PCR patterns, we found that 14 isolates were included in six genetic groups: GgII (isolates 9 and 10), GgQ (isolates 18 and 20), GgDD (isolates 30–33), and GgEE (isolates 38 and 40) for *S. aureus;* GgD (isolates 48 and 49) for *B. cereus*; and GgVI (isolates 58 and 59) for *E. f*ae*calis*—indicating that (i) these isolates were involved in the translocation process and (ii) the isolates within each Gg were clonally related.

## Discussion

Although molecular typing has often been used to characterized BT in a clinical setting [24, 25], there are few literature data on the use of these techniques to detect translocation from the intestinal tract in animal models [26]. To the best of our knowledge, the present study is the first to have employed molecular typing (ERIC2-PCR and RAPD-PCR) techniques in the detection of BT in the rat. We observed BT during critical life periods in 22.5 % of the studied CPF-exposed rats. In studies of rats with ascites and carbon-tetrachloride-induced cirrhosis, the prevalence of BT ranged from 36.8 to 97.6 % [27–30]. In a clinical study, positive MLN cultures were found for 32.1 % of patients with cirrhosis [31].

These findings indicate that the prevalence of BT depends on the type of study and the detection methods used (bacteriological culture of MLN samples). It has been reported that bacterial DNA in the biological fluids is a marker for BT in rats with cirrhosis [28, 31]; this suggests that the definition of BT has been widened to the passage of bacterial fragments (endotoxins, bacterial DNA, etc.) from the intestinal lumen to the MLNs and into the circulatory system in general. Although they came from the same species, translocated strains and non-translocated strains differed clearly in terms of the ERIC2-PCR and RAPD-PCR patterns (77.5 % of strains). As reported in the literature [26], we concluded that BT did not occur in samples when the extra-intestinal organs (liver, spleen, Peyer’s patches, MLNs, adipose tissue, and kidneys) were sterile. One could argue that the intestinal bacteria found in the extra-intestinal tissue samples resulted from bacterial contamination and not from BT. Table 3 shows that 41.5 % (22 out of 53) of the isolates in the extra-intestinal tissues were not found in intestinal tissues. Conversely, 58.5 % (31 out of 53) of the isolates in intestinal tissues were absent from extra-intestinal samples. This discrepancy might have been due to slight differences between the intestinal bacterial counts in rats with BT vs. rats without BT. Our results contradicts the literature data on BT in animal models, in which bacterial overgrowth was identified as a major BT-promoting factor [32]. In rats, anaerobic bacteria predominate in the ileum, caecum and colon. This anaerobic flora rarely translocates, and thus limits the colonization and growth of potentially translocating bacteria. The intestinal mucosa thus constitutes a barrier against BT via the transcellular route (through enterocytes) or the paracellular route (at tight junctions). Studies in animals have shown that some antibiotics (such as norfloxacin and trimethoprim–sulfamethoxazole) selectively reduce intestinal bacterial overgrowth (and thus BT) by changing the composition of the bacterial flora. In a cirrhotic rat model, the administration of propranolol or cisapride stimulated intestinal motility by speeding up intestinal transit, decreasing bacterial overgrowth, improving intestinal permeability and reducing BT. Similarly, the administration of conjugated biliary acids (cholylsarcosine and cholylglycine) suppressed intestinal bacterial overgrowth and reduced BT. Lastly, the use of antioxidants alone or in combination with probiotics (*Lactobacillus*) in cirrhotic rats reduced the intestinal bacterial load, BT, and oxidative stress in the intestinal wall.

Several previous studies have investigated the effect of CPF on the intestinal wall (ileum and colon) [4, 5]. Exposure to CPF decreases the mRNA expression of genes encoding the tight junction proteins (including claudin-4 and zonula-1); this increases intestinal permeability, enables BT and activates the immune system. In the present study, all the bacterial strains isolated from individual samples from CPF-exposed rats were found to be viable on agar plates, which suggests that the strains tolerated CPF doses of 1 mg/kg BW and 5 mg/kg BW in vivo. All of the isolates were then tested for their susceptibility to 20 antibiotics. Over 90 % of the detected bacterial strains were susceptible; 2 strains (5 %) of *S. aureus* expressed the MRSA phenotype (PEN^r^ OXA^r^ FOX^r^) but were susceptible to the remaining antibiotics, 9 isolates (45 %) of *E. faecalis* were resistant to Er, 6 isolates (30 %) were resistant to PT, and 100 % of the isolates were resistant to L (natural resistance). For *E. faecalis*, 3 isolates (15 %) expressed the macrolide-lincosamide-streptogramin B (MLSB) + streptogramin A (SA) phenotype (Er^r^L^r^PT^r^), and 12 isolates (60 %) expressed the MLSB phenotype (Er^r^L^r^PT^s^). In strains expressing the MLSB phenotype, the genes encoding resistance are located on a 20–30 kb plasmid [33]. However, resistance is chromosome-encoded in the majority of strains isolated in France [33]. Resistance to macrolide, lincosamide, SA (PT) and SB (virginiamycin) is associated with the production of an SA-inactivating mutant acetyltransferase and an SB-inactivating mutant hydrolase [34–36]. Expression of the MRSA phenotype and susceptibility to other antibiotics has been documented in the literature [17, 37–40].

Methicillin resistance is associated with a specific mechanism in which a mobile genetic element [the staphylococcal cassette chromosome (SCC) *mec*] integrates into the *S. aureus* chromosome. In the *S. aureus* chromosome, the *mec A* gene encodes a specific, methicillin-resistant transpeptidase (penicillin-binding protein 2a) [38], which results in resistance to all β-lactam antibiotics (due to a low affinity for binding to β-lactam). The classic *mecA* gene codes for resistance to methicillin, kanamycin, gentamicin (in most cases), tobramycin, amikacin, Er, and L but not for resistance to PT and OFX. A new *mecA* homolog *mecB* strain (N315) [37, 38] and a *mecC* strain (LGA256) [39, 40] have been identified. The SCC elements classified as *mecB* or *mecC* share ≥70 % nucleotide sequence identity with the classic *mecA* gene. In the present study, the isolates were phenotypically resistant to all β-lactams but remained susceptible to other tested antibiotics and thus corresponded chromosomally to the *mecC* gene. Our results are in agreement with the literature data [17, 37–40]. When considering the genotyped strains, resistant strains (*B. cereus* and *E. faecalis*) were only found in the CPF groups (both CPF1 and CPF5)—particularly in juvenile rats at D21. Naphade et al. [41] detected 9.8 and 26.2 kDa plasmids in bacterial cultures from garden soil, indicating that the ability to tolerate high concentrations of heavy metal salts, pesticides and antibiotics is a plasmid-encoded characteristic. Harishankar et al. [42] study of five model intestinal bacteria found that *Lactobacillus fermentum*, *Lactobacillus lactis*, and *Escherichia coli* tolerated high concentrations of CPF (>1400 µg/mL), whereas *E. faecalis* and *Lactobacillus plantarum* tolerated lower concentrations (400 and 100 μg/mL, respectively).

The three most tolerant bacteria (*L. fermentum*, *L. lactis*, and *E. coli*) produced an organophosphorus phosphatase that degrades CPF; the concentration of CPF was higher outside the cells (i.e. in the supernatant) than inside the cells. *L. fermentum* degraded 70 % of the CPF (with 3,5,6-trichloro-2-pyridinol as the end product), *L. lactis* degraded up to 61 % of the CPF (with CPF oxon as the end product), whereas *E. coli* degraded only 16 % of the CPF (with CPF oxon and diethylphosphate as the end products). This microbial variation in pesticide tolerance was dependent on the end-product degradation profiles and/or the types of end products [43, 44]. Shafiani and Malik [44] reported that a *Pseudomonas* spp. isolated from soil was able to tolerate up to 800 μg/mL of endosulfan, 1600 μg/mL of carbofuran and 1600 μg/mL of malathion. All of the bacterial isolates were further tested for their antibiotic susceptibility to seven different antibiotics. A bacteriological analysis of endosulfan-exposed soil by Sepperumal et al. [45] revealed the presence of *Bacillus ciradans*, *Pseudo*monas spp., *Bacillus lentus*, *Acinetobacter* spp., and *B. cereus*. Additional plasmid-curing experiments were performed to determine whether pesticide resistance traits in these bacteria were encoded on a plasmid or on the bacterial chromosome. Furthermore, *B. ciradans* and *Acinetobacter* spp. contained plasmids of 7 and 4 kb in size, respectively. Once BT has occurred, the isolation of viable bacteria in the liver, spleen, adipose tissue, MLNs and kidneys depends on (i) the immunological competence of the host and (ii) individual bacterial virulence factors that prevent the pathogen’s destruction [46].

We were unable to isolate anaerobic bacteria from the *Enterobacteriace*ae family; this constitutes a study limitation. In holoxenic rats, the stomach and bowel is mainly colonized from birth to weaning by *Lactobacillus*, *Streptococcus* and *Enterococcus* genera, where as *Enterobacteriace*ae and anaerobic bacteria are absent [47, 48]. After weaning, the pups’ bacterial flora downstream of the ileum and caecum predominantly consists of anaerobic bacteria [49]. These results are consistent with Raybaud et al. [44] observation of the absence of the *Enterobacteriace*ae family. However, our failure to isolate anaerobic bacteria was probably due to the detection technique used.

In conclusion, our earlier morphological and molecular analyses showed that long-lasting exposure to CPF (an organophosphate insecticide observed as food residues) altered the maturation of the rat intestine and was associated with intestinal dysbiosis and BT towards sterile organs [4, 5]. In the present study, we confirmed the occurrence of BT and validated the use of molecular typing in rats exposed to CPF in utero and during lactation.

The present study showed that molecular typing can detect intestinal BT in CPF-exposed rats; a combination of ERIC2-PCR and RAPD-PCR patterns can be used as a DNA fingerprint for intestinal microfloral strains. The most prevalent bacteria in intestinal samples from CPF-exposed rats were *S. aureus*, *E. faecalis* and *B. cereus*. Furthermore, chromosome- and plasmid-encoded pesticide-tolerance and antibiotic-resistance traits were observed in *S. aureus* and *E. faecalis* strains.

Lastly, we believe that our present findings provide strong evidence to show that early in utero and lactational exposure to CPF may have short- and long-lasting impacts on the digestive system. Importantly, CPF is found in cereals, fruit, vegetables and (potentially) in meat and drinking water, and is known to cross the placental barrier. Under normal circumstances, the intestinal epithelium acts as a barrier against food antigens and commensal bacteria. The latter bacteria live in symbiosis with the host and are significantly involved in physiological functions. The present data on gut homeostasis indicate that CPF disrupts the gut maturation, alters the bacterial equilibrium and enhances BT towards sterile organs. Moreover, the observed effect may be even greater during the food diversification period that occurs after weaning. Hence, we strongly believe that pesticide exposure in infancy (when the digestive system is still immature) should be avoided as much as possible.

## Abbreviations

BT: bacterial translocation
BW: bodyweight
CI: confidence interval
CPF: chlorpyrifos
CPF0: control group
D: day
Er: erythromycin
ERIC2: enterobacterial repetitive intergenic consensus 2
Gg: genetic groups
I: intermediate
FOX: cefoxitin
L: lincomycin
MALDI–TOF-MS: matrix-assisted laser desorption/ionization time-of-flight mass spectrometry
MLN: mesenteric lymph nodes
MLSB: macrolide-lincosamide-streptogramin B
MRSA: methicillin-resistance *Staphylococcus aureus*
OFX: ofloxacin
OR: odds ratio
OXA: oxacillin
PCR: polymerase chain reaction
PEN: penicillin
PT: pristinamycin
R: resistant
RAPD: random amplification polymorphic DNA
SCC: staphylococcal cassette chromosome
S: susceptible
SA: streptogramin
